# Population‐level evidence of survival benefits of patient‐reported outcome symptom monitoring software systems in routine cancer care

**DOI:** 10.1002/cam4.3480

**Published:** 2020-10-07

**Authors:** Ethan Basch, Marjory Charlot, Amylou C. Dueck

**Affiliations:** ^1^ Lineberger Comprehensive Cancer Center University of North Carolina Chapel Hill NC USA; ^2^ Division of Oncology University of North Carolina Chapel Hill NC USA; ^3^ Mayo Clinic Scottsdale AZ USA

**Keywords:** behavioral science, QOL, quality of life, survival

Symptom management is a cornerstone of quality oncology care. Most patients with cancer experience debilitating symptoms related to the disease itself or to treatment toxicities.^1^ If symptoms are not detected early enough, they can worsen and lead to unnecessary suffering, avoidable hospital services, and even death.^2^ Numerous prior studies have shown that up to half of patients' symptoms go undetected by their care teams.^3^, ^4^, ^5^ There is therefore a substantial opportunity to improve symptom detection and early interventions to avoid downstream complications and improve outcomes.

Symptom monitoring via electronic patient‐reported outcomes (PROs) is one strategy for detecting problems and conveying them to care teams, with a substantial body of research spanning two decades, particularly in populations with metastatic cancers.^6^, ^7^, ^8^, ^9^, ^10^, ^11^ The general approach includes a brief (10‐15 item) questionnaire loaded into a software system via which patients may self‐report by web, downloadable application, or automated telephone interface on a regular basis. Patients may self‐report at clinic visits via tablet computers or kiosks, and may report from home between visits using their own devices. Automated alerts are triggered to the patient's care team whenever a worsening or severe symptom is reported, to inform interventions. Often, a core group of common, cross‐cutting symptoms is included^12^; additional tailored items specific to a cancer type or treatment type may be added; and an open‐ended free‐text item may also be included.^13^ Items about other issues of concern such as physical function (i.e., performance status), or financial burden, are increasingly included in these PRO monitoring systems.

A host of clinical and utilization benefits has been demonstrated with implementation of such PRO monitoring systems in routine cancer care, leading them to be classified as “digital therapeutics” recently. Benefits of PRO monitoring systems in prior clinical research include improved symptom detection and control; patient satisfaction; patient‐clinician communication; quality of life; physical functioning; increased duration of chemotherapy treatment; decreased emergency room and hospital use^6^, ^7^, ^8^, ^9^—and most recently two prospective randomized trials showing overall survival benefits.^10^, ^11^

Missing from the literature has been a large, real‐world study evaluating the clinical impact of PRO symptom monitoring. In this issue of *Cancer Medicine*, Barbera and colleagues provide such evidence.^14^ They report on a population‐level implementation of PRO monitoring in the Canadian province of Ontario, where since 2007 the Edmonton Symptom Inventory System (ESAS) has been offered to patients for completion via computer kiosks at oncology clinic visits. This retrospective analysis included adults diagnosed with cancer between 2007 and 2015. The authors linked PRO data to the provincial cancer registry at the patient level, enabling evaluation of administrative and clinical information. They included patients who had completed at least one PRO self‐report at an outpatient visit. A comparator group of patients who did not complete PROs was generated using hard and propensity score matching.

The authors identified 128,893 matched patient pairs for analysis. In the PRO group, there was a median of 3 completed ESAS assessments. Five year survival was significantly greater in the PRO group than the comparator group (81.9% vs 76.4% at 1 year; 68.3% vs 66.1% at 3 years; 61.9% vs 61.4% at 5 years; *p* < 0.0001). The observed benefit at 1 year with PRO symptom monitoring of a 5.5% absolute overall survival benefit exceeds the benefits of many existing cancer drugs—and with substantially less “toxicity.”

This study adds to existing research, and there is now evidence from both randomized controlled trials and population‐level assessment that there is a survival benefit associated with electronic PRO symptom monitoring. Although the study by Barbera et al is limited by not being prospective or randomized, the authors employed rigorous observational research methods to balance and match groups. The focus of the Ontario PRO program they describe has been on in‐clinic reporting, but greater opportunities lie in expansion of this approach to incorporate remote monitoring of patients between visits. This has been the approach used in other PRO programs, and hopefully is the direction Ontario will be taking as well.

Future research efforts should focus on challenges and strategies related to effective implementation of PROs in real‐world clinical settings, and development of standardized recommendations. Each institution implementing PROs will need to adopt approaches that optimize engagement and buy‐in of their patients, clinicians, and staff members. This will likely require reorganization of workflow and personnel deployment.

For patient users, implementation will ideally involve assuring that the technology itself is easy to use and meaningful, with continuous reminders to them that using the system is an essential part of care. For clinician users, the technology must also be easy to use, and integrated into existing systems and workflows. It must be reinforced by institutional leadership to clinicians that this is an integral part of care. For clinicians who field incoming PRO alerts, adequate time must be allotted for them to address alerts, rather than simply adding this work on top of existing responsibilities.

PRO interfaces to date have been fairly rudimentary, with static questionnaires for patients, and wrote reports for clinicians. There are opportunities to enable these interfaces to become more interactive and engaging, for example, by using chatbot conversational approaches, gamification, and human factors methods.

Equity is another key area where implementation research is warranted. As remote monitoring becomes increasingly integrated into routine care processes, there is a risk that those without technical literacy or with limited internet access could be left behind. In other words, the digital divide could become translated into greater heath disparities. Rather, patient‐facing technologies like PRO interfaces should be tools to narrow disparities. To do so, attention must be given to the hardware patients use to access systems, and to the usability of systems. For example, in a large ongoing PRO implementation study in the United States, about 40% of patients selected to use an automated telephone system rather than web to self‐report symptoms.^15^ This illustrates the importance of meeting patients where they are, and offering interfaces that they are willing and able to use.

Finally, in the future, a reimbursement model for supporting use of PRO symptom monitoring in oncology is needed. Since PRO monitoring reduces emergency and hospital services, lengthens time on treatment, improves patient experience and lengthens survival, it is reasonable to look to payers for support. In the case of the Ontario PRO program described by Barbera and colleagues, indeed the program is wrapped into the Provincial financial model for care delivery. In the United States, the Medicare program may provide some support for PRO implementation as part of its pending Oncology Care First payment model.^16^ Medicare should also provide or expand a billing code to enable payment for PRO symptom monitoring in the fee‐for‐service environment, and private payers in the US and non‐US payment mechanisms should follow suit.

As telemedicine becomes a standard part of how we practice medicine in the era of COVID‐19, patients will increasingly be “out of sight and out of mind” between in‐person encounters. Remote monitoring via PROs brings an opportunity to improve how we continuously engage and assure the health of our patients, and should become a cornerstone of population health management. Ontario serves as a model for other institutions, showing that connecting with patients using novel technologies can increase engagement while improving outcomes.

## CONFLICT OF INTEREST

Dr. Basch receives payments as a scientific advisor to CareVive, Navigating Cancer, AstraZeneca, and Sivan. He receives research funding to his institution from the National Cancer Institute and the Patient‐Centered Outcomes Research Institute.

## AUTHOR CONTRIBUTION

All authors of this editorial have met the following criteria: 1. Have made substantial contributions to conception and design, and interpretation; 2. Been involved in drafting the manuscript and revising it critically for important intellectual content; 3. Given final approval of the version to be published and participated sufficiently in the work to take public responsibility for appropriate portions of the content; and 4. Agreed to be accountable for all aspects of the work in ensuring that questions related to the accuracy or integrity of any part of the work are appropriately investigated and resolved.

## Data Availability

This is an editorial and there is no primary data or data analysis related to this editorial.
